# Development of a Machine Learning Model for Distant Metastasis Risk Stratification in Acral Melanoma

**DOI:** 10.1002/cnr2.70569

**Published:** 2026-05-24

**Authors:** Ye Shanyuan, Zhang Rundong, Cao Meng, Qiu Zequn, Zhang Qian, Lv Qun, Wang Yan

**Affiliations:** ^1^ Hospital for Skin Diseases, Institute of Dermatology, Chinese Academy of Medical Sciences & Peking Union Medical College Nanjing Jiangsu China

**Keywords:** acral melanoma, distant metastasis, LightGBM, machine learning, prediction model

## Abstract

**Background:**

Acral melanoma (AM) is a distinct melanoma subtype associated with delayed diagnosis, aggressive progression, and poor prognosis once distant metastasis occurs. However, prediction models specifically designed for distant metastasis risk stratification in AM remain limited.

**Aims:**

This study aimed to develop and internally evaluate a machine learning‐based model for individualized distant metastasis risk stratification in patients with AM.

**Methods and Results:**

Clinical data of 1822 patients with AM diagnosed between 2000 and 2021 were extracted from the SEER database. Patients were divided into training and internal test sets at a ratio of 7:3 using stratified sampling. Logistic regression analyses were performed to identify factors associated with distant metastasis, and six machine learning algorithms were developed and compared. SMOTE was applied only to the training set to address class imbalance. Multivariate logistic regression identified sentinel lymph node biopsy as an independent protective factor, whereas higher N stage and lower median household income were independent risk factors. Among the evaluated models, LightGBM showed relatively balanced overall performance and was selected as the optimal model. SHAP analysis identified N stage, sentinel lymph node biopsy record, and median household income as the most important predictors.

**Conclusion:**

The LightGBM model demonstrated moderate predictive performance for distant metastasis risk stratification in patients with AM. This model may serve as a research‐oriented tool for individualized risk assessment, although external validation using independent real‐world cohorts is required before clinical application.

## 1 Introduction

Melanoma most commonly arises in sun‐exposed cutaneous sites, whereas acral melanoma (AM) is a distinct subtype that occurs on the palms, soles, and nail apparatus and is not strongly associated with ultraviolet exposure. This subtype is more prevalent among Asian and African populations, whereas its incidence is relatively low in Caucasians [1, 2]. Despite its lower overall incidence, AM is characterized by delayed diagnosis and advanced presentation, often leading to worse prognoses compared to cutaneous melanoma [3]. The pathways and mechanisms underlying the metastatic spread of AM are not fully understood, but evidence suggests that the tumor's unique microenvironment, vascularization patterns, and interactions with the immune system play critical roles [4]. Common sites of distant metastasis include the lungs, liver, brain, and bones, with metastasis often occurring earlier and more aggressively in AM compared to other melanoma subtypes [5]. In addition to clinically detectable distant metastasis, the presence of micrometastasis, which cannot be identified through routine diagnostic tests, significantly worsens the prognosis of patients [6]. Therefore, the ability to predict the risk of distant metastasis is critical. Early identification of metastasis risk helps tailor personalized treatment plans for patients, thereby improving survival rates and optimizing treatment outcomes.

Machine learning and artificial intelligence techniques have been increasingly adopted in medical clinical decision‐support systems [7, 8, 9], enabling more accurate and personalized care. Chan et al. utilized six machine learning algorithms to develop and validate a metastasis and prognosis model for patients with nodular melanoma [10]. In addition, Wu et al. developed a uveal melanoma distant metastasis prediction system based on machine learning [11]. These models can assess distant metastasis risk, predict overall survival (OS), and support personalized treatment and follow‐up strategies. A key aspect of these systems involves the development of clinical prediction models, which typically require training on large datasets and subsequent validation via test sets [12]. Despite these advancements, research on metastasis in AM patients remains sparse, with a significant gap in predictive models to guide clinical decision‐making. To address this gap, we utilized the SEER database (2000–2021) to clean and analyze data, aiming to predict the risk of distant metastasis among patients with AM. This study aimed to advance precision medicine by constructing a novel predictive model tailored to this underexplored area.

## 2 Materials and Methods

### 2.1 Screening and Enrollment Process

**FIGURE 1:**
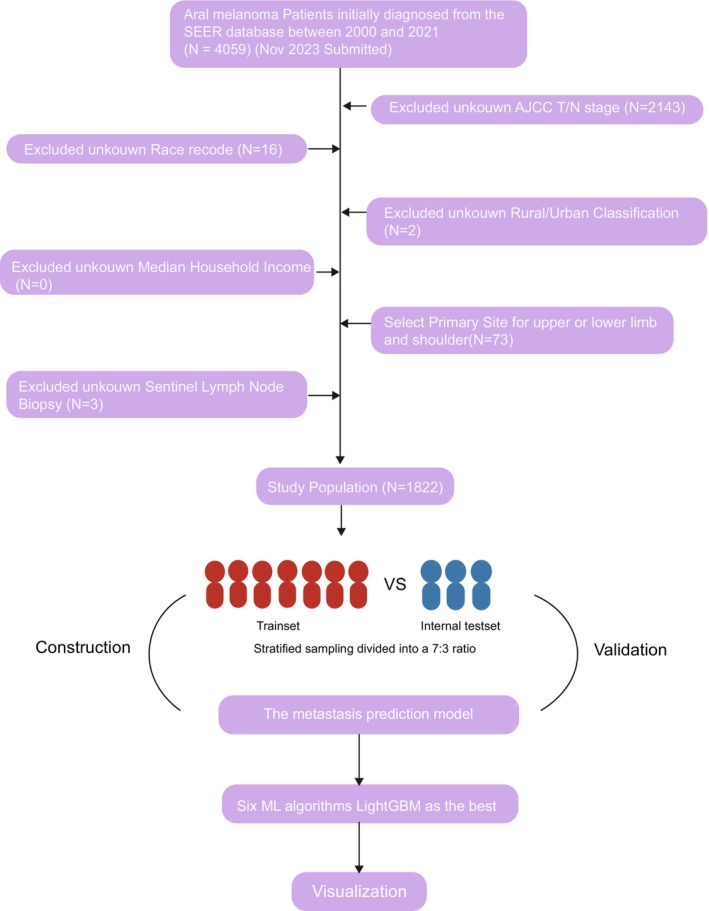
Flowchart of the data cleaning process for patients with acral melanoma in the SEER database.

We retrieved clinical information of 1822 patients with AM from the SEER database, including cases diagnosed from 2000 to 2021, and following the specified inclusion and exclusion criteria (Figure 1). Data extraction was performed independently by two investigators. When there were any differences, the third investigator was consulted. All SEER data were anonymized, and patient privacy was protected. Because the SEER database contains publicly available de‐identified data, informed consent was not required.

### 2.2 Data Selection

During data preprocessing, the following variables were retained for analysis: age, sex, race, total number of malignant tumors, rural–urban continuum code, inflation‐adjusted median household income, interval from diagnosis to treatment, primary tumor site, sentinel lymph node biopsy record, chemotherapy recoding, systemic therapy recoding, surgery records, AJCC T stage, and N stage.

### 2.3 Handling of Class Imbalance

To address class imbalance in the training dataset, the Synthetic Minority Over‐sampling Technique (SMOTE) was applied. Categorical variables were transformed into numerical features using one‐hot encoding prior to resampling. SMOTE generates synthetic minority‐class samples by interpolating between nearest neighbors in the feature space, thereby reducing the impact of imbalanced data distribution. All resampling procedures were conducted exclusively on the training set to avoid information leakage, while the test set was retained for independent performance evaluation.

### 2.4 Machine Learning Model Development

The dataset was randomly divided into a training set and an internal test set at a ratio of 7:3 using stratified sampling based on the outcome variable (*meta*). Data preprocessing was performed within the training set only. Cases with missing predictor values were excluded using complete‐case analysis (step_naomit(all_predictors(), skip = FALSE)), and no imputation was performed. Nominal predictors were converted into dummy variables using step_dummy(all_nominal_predictors()).

To address class imbalance, SMOTE was applied only to the training set using step_smote(meta, over_ratio = 1), yielding an approximately balanced class distribution. The internal test set was kept unchanged to avoid information leakage and to preserve the original outcome distribution.

The LightGBM model was implemented using the tidymodels and bonsai framework. Six hyperparameters (tree_depth, trees, learn_rate, mtry, min_n, and loss_reduction) were tuned by random search with 10‐fold cross‐validation. The search ranges were as follows: tree_depth, 1–3; trees, 100–500; learn_rate, 0.001–0.1; mtry, 2–8; min_n, 5–10; and loss_reduction, 0.001–1. The optimal hyperparameter combination was selected primarily based on ROC AUC. The final model was then fitted on the training set and evaluated on the internal test set. Accuracy was additionally reported as a supplementary performance metric.

### 2.5 Development and Validation of the Distant Metastasis Prediction Model

Univariate and multivariate logistic regression analyses were performed to identify independent risk factors for AM metastasis. To ensure consistency in the distribution of the target variable (AM metastasis) between the training and test sets, stratified sampling was applied to split the dataset into a training set (70%) and a test set (30%). Six machine learning algorithms, including LightGBM [13], elastic net (ENet) [14], XGBoost [15], multilayer perceptron (MLP) [16], random forest (RF) [17], and logistic regression [18], were used to develop predictive models for AM metastasis. Model performance was evaluated via 10‐fold cross‐validation, radar charts, and confusion matrix analysis [19]. The LightGBM demonstrated better predictive performance and was selected as the optimal model. Key predictors of AM metastasis were identified through feature importance analysis, and a web‐based calculator was developed to facilitate model visualization and risk assessment.

### 2.6 Model Interpretation

Based on cooperative game theory, SHAP provides interpretability for complex black‐box machine learning models [20]. This method quantifies the contribution of each feature to the predicted risk of distant metastasis. In SHAP, the factors with positive SHAP values indicated by yellow are risk factors for distant metastasis in AM patients. Factors with negative SHAP values indicated by purple are protective factors. We used the LightGBM model, which was identified as the optimal predictive model for distant metastasis in AM patients and ranked features from highest to lowest. Two additional patients were randomly selected to provide personalized model explanations and illustrate their predicted risk of distant metastasis.

### 2.7 Statistical Analysis

All the statistical analyses were carried out using R (version 4.2.0). Machine learning models were developed and fine‐tuned on the training set, which was obtained via stratified sampling to split the dataset into a 7:3 ratio for training and testing. Model accuracy was evaluated via the internal test set. Continuous variables were compared using the *t*‐test, whereas categorical variables were compared using the chi‐square test. The machine learning models and the web‐based calculator were developed using the R package Shiny. A *p* value less than 0.05 was regarded as statistically significant.

## 3 Results

### 3.1 Patient Characteristics

The dataset from the SEER database included melanoma patients from 2000 to 2021, with a total of 1822 eligible AM patients selected based on the inclusion and exclusion criteria. Among these patients, 874 (48.0%) were male, and 948 (52.0%) were female, with 83.6% identified as Caucasian (Table 1). Patients were divided into metastatic and nonmetastatic groups, and chi‐square tests were performed for each variable. The results revealed statistically significant differences (*p* < 0.05) between the metastatic and nonmetastatic groups across six variables: total number of malignant tumors, sentinel lymph node biopsy records, chemotherapy recoding, systemic therapy recoding, T stage, and N stage (Table 1). Table 2 presents the baseline characteristics of patients with and without distant metastasis in the training and internal test sets. The variables chemotherapy recording, systemic therapy recording, T stage, and N stage were both in the significant difference group (*p* < 0.05) when comparing the distant metastasis group and the no distant metastasis group in both the training set and the internal test set. Sentinel lymph node biopsy records also showed a significant difference (*p* < 0.05).

**TABLE 1:** Patient baseline information.

Variables	No distant metastasis (*N* = 1777)	Distant metastasis (*N* = 45)	Overall (*N* = 1822)	*p*
Age				
> 60	962 (54.1%)	20 (44.4%)	982 (53.9%)	0.256
≤ 60	815 (45.9%)	25 (55.6%)	840 (46.1%)	
Sex				
Female	929 (52.3%)	19 (42.2%)	948 (52.0%)	0.237
Male	848 (47.7%)	26 (57.8%)	874 (48.0%)	
Race				
Black	137 (7.7%)	4 (8.9%)	141 (7.7%)	0.491
Others (American Indian/Alaska Native, Asian or Pacific Islander)	152 (8.6%)	6 (13.3%)	158 (8.7%)	
White	1488 (83.7%)	35 (77.8%)	1523 (83.6%)	
Total number of malignant tumors				
> 1	632 (35.6%)	9 (20.0%)	641 (35.2%)	0.045
1	1145 (64.4%)	36 (80.0%)	1181 (64.8%)	
Rural–urban continuum code				
Metropolitan	1564 (88.0%)	37 (82.2%)	1601 (87.9%)	0.345
Nonmetropolitan	213 (12.0%)	8 (17.8%)	221 (12.1%)	
Median household income inflation adj to 2022				
$60 000–$89 999	1016 (57.2%)	22 (48.9%)	1038 (57.0%)	0.051
≤ $60 000	275 (15.5%)	13 (28.9%)	288 (15.8%)	
≥ $90 000	486 (27.3%)	10 (22.2%)	496 (27.2%)	
Time from diagnosis to treatment				
0–7 days	901 (50.7%)	28 (62.2%)	929 (51.0%)	0.333
31–90 days	347 (19.5%)	7 (15.6%)	354 (19.4%)	
8–30 days	326 (18.3%)	4 (8.9%)	330 (18.1%)	
Over 91 days	40 (2.3%)	2 (4.4%)	42 (2.3%)	
Unable to calculate	163 (9.2%)	4 (8.9%)	167 (9.2%)	
Primary site				
Skin of upper limb and shoulder	331 (18.6%)	6 (13.3%)	337 (18.5%)	0.478
Skin of lower limb and hip	1446 (81.4%)	39 (86.7%)	1485 (81.5%)	
Sentinel lymph node biopsy record				
No sentinel biopsy	889 (50.0%)	34 (75.6%)	923 (50.7%)	0.001
Performed sentinel biopsy	888 (50.0%)	11 (24.4%)	899 (49.3%)	
Chemotherapy recode				
No	1732 (97.5%)	37 (82.2%)	1769 (97.1%)	< 0.001
Yes	45 (2.5%)	8 (17.8%)	53 (2.9%)	
Systemic therapy recode				
No	1275 (71.8%)	21 (46.7%)	1296 (71.1%)	< 0.001
Yes	502 (28.2%)	24 (53.3%)	526 (28.9%)	
Surgery record				
No	43 (2.4%)	3 (6.7%)	46 (2.5%)	0.189
Yes	1734 (97.6%)	42 (93.3%)	1776 (97.5%)	
T stage				
T1	653 (36.7%)	6 (13.3%)	659 (36.2%)	0.001
T2	379 (21.3%)	9 (20.0%)	388 (21.3%)	
T3	408 (23.0%)	10 (22.2%)	418 (22.9%)	
T4	337 (19.0%)	20 (44.4%)	357 (19.6%)	
N stage				
N0	1383 (77.8%)	15 (33.3%)	1398 (76.7%)	0.001
N1	184 (10.4%)	14 (31.1%)	198 (10.9%)	
N2	144 (8.1%)	6 (13.3%)	150 (8.2%)	
N3	66 (3.7%)	10 (22.2%)	76 (4.2%)	

**TABLE 2:** Comparison of baseline data for distant metastasis between the training set and internal test set.

Variables	Train set			*p*	Internal test set			*p*
	Distant metastasis (*N* = 33)	No distant metastasis (*N* = 1242)	Overall (*N* = 1275)		Distant metastasis (*N* = 12)	No distant metastasis (*N* = 535)	Overall (*N* = 547)	
Age								
> 60	13 (39.4%)	666 (53.6%)	679 (53.3%)	0.15	7 (58.3%)	296 (55.3%)	303 (55.4%)	1
≤ 60	20 (60.6%)	576 (46.4%)	596 (46.7%)		5 (41.7%)	239 (44.7%)	244 (44.6%)	
Sex								
Female	15 (45.5%)	656 (52.8%)	671 (52.6%)	0.51	4 (33.3%)	273 (51.0%)	277 (50.6%)	0.357
Male	18 (54.5%)	586 (47.2%)	604 (47.4%)		8 (66.7%)	262 (49.0%)	270 (49.4%)	
Race								
Black	4 (12.1%)	93 (7.5%)	97 (7.6%)	0.465	2 (16.7%)	42 (7.9%)	44 (8.0%)	0.346
Others (American Indian/Alaska Native, Asian or Pacific Islander)	4 (12.1%)	110 (8.9%)	114 (8.9%)		10 (83.3%)	449 (83.9%)	459 (83.9%)	
White	25 (75.8%)	1039 (83.7%)	1064 (83.5%)		0 (0%)	44 (8.2%)	44 (8.0%)	
Total number of malignant tumors								
> 1	7 (21.2%)	435 (35.0%)	442 (34.7%)	0.144	2 (16.7%)	197 (36.8%)	199 (36.4%)	0.258
1	26 (78.8%)	807 (65.0%)	833 (65.3%)		10 (83.3%)	338 (63.2%)	348 (63.6%)	
Rural–urban continuum code								
Metropolitan	27 (81.8%)	1096 (88.2%)	1123 (88.1%)	0.394	10 (83.3%)	468 (87.5%)	478 (87.4%)	1
Nonmetropolitan	6 (18.2%)	146 (11.8%)	152 (11.9%)		2 (16.7%)	67 (12.5%)	69 (12.6%)	
Median household income inflation adj to 2022								
$60 000–$89 999	17 (51.5%)	697 (56.1%)	714 (56.0%)	0.138	5 (41.7%)	319 (59.6%)	324 (59.2%)	0.276
≤ $60 000	9 (27.3%)	186 (15.0%)	195 (15.3%)		4 (33.3%)	89 (16.6%)	93 (17.0%)	
≥ $90 000	7 (21.2%)	359 (28.9%)	366 (28.7%)		3 (25.0%)	127 (23.7%)	130 (23.8%)	
Time from diagnosis to treatment								
0–7 days	18 (54.5%)	642 (51.7%)	660 (51.8%)	0.648	10 (83.3%)	259 (48.4%)	269 (49.2%)	0.158
31–90 days	6 (18.2%)	227 (18.3%)	233 (18.3%)		1 (8.3%)	120 (22.4%)	121 (22.1%)	
8–30 days	4 (12.1%)	220 (17.7%)	224 (17.6%)		1 (8.3%)	39 (7.3%)	40 (7.3%)	
Over 91 days	2 (6.1%)	29 (2.3%)	31 (2.4%)		0 (0%)	106 (19.8%)	106 (19.4%)	
Unable to calculate	3 (9.1%)	124 (10.0%)	127 (10.0%)		0 (0%)	11 (2.1%)	11 (2.0%)	
Primary site								
Skin of upper limb and shoulder	5 (15.2%)	233 (18.8%)	238 (18.7%)	0.765	1 (8.3%)	98 (18.3%)	99 (18.1%)	0.61
Skin of lower limb and hip	28 (84.8%)	1009 (81.2%)	1037 (81.3%)		11 (91.7%)	437 (81.7%)	448 (81.9%)	
Sentinel lymph node biopsy record								
No sentinel biopsy	24 (72.7%)	618 (49.8%)	642 (50.4%)	0.015	10 (83.3%)	271 (50.7%)	281 (51.4%)	0.051
Performed sentinel biopsy	9 (27.3%)	624 (50.2%)	633 (49.6%)		2 (16.7%)	264 (49.3%)	266 (48.6%)	
Chemotherapy recode								
No	29 (87.9%)	1206 (97.1%)	1235 (96.9%)	0.013	8 (66.7%)	526 (98.3%)	534 (97.6%)	< 0.001
Yes	4 (12.1%)	36 (2.9%)	40 (3.1%)		4 (33.3%)	9 (1.7%)	13 (2.4%)	
Systemic therapy recode								
No	16 (48.5%)	874 (70.4%)	890 (69.8%)	0.012	5 (41.7%)	401 (75.0%)	406 (74.2%)	0.023
Yes	17 (51.5%)	368 (29.6%)	385 (30.2%)		7 (58.3%)	134 (25.0%)	141 (25.8%)	
Surgery record								
No	2 (6.1%)	31 (2.5%)	33 (2.6%)	0.473	1 (8.3%)	12 (2.2%)	13 (2.4%)	0.681
Yes	31 (93.9%)	1211 (97.5%)	1242 (97.4%)		11 (91.7%)	523 (97.8%)	534 (97.6%)	
T stage								
T1	3 (9.1%)	470 (37.8%)	473 (37.1%)	0.002	3 (25.0%)	183 (34.2%)	186 (34.0%)	0.016
T2	8 (24.2%)	280 (22.5%)	288 (22.6%)		1 (8.3%)	99 (18.5%)	100 (18.3%)	
T3	9 (27.3%)	265 (21.3%)	274 (21.5%)		1 (8.3%)	143 (26.7%)	144 (26.3%)	
T4	13 (39.4%)	227 (18.3%)	240 (18.8%)		7 (58.3%)	110 (20.6%)	117 (21.4%)	
N stage								
N0	10 (30.3%)	964 (77.6%)	974 (76.4%)	< 0.001	5 (41.7%)	419 (78.3%)	424 (77.5%)	0.002
N1	10 (30.3%)	125 (10.1%)	135 (10.6%)		4 (33.3%)	59 (11.0%)	63 (11.5%)	
N2	5 (15.2%)	102 (8.2%)	107 (8.4%)		1 (8.3%)	42 (7.9%)	43 (7.9%)	
N3	8 (24.2%)	51 (4.1%)	59 (4.6%)		2 (16.7%)	15 (2.8%)	17 (3.1%)	

### 3.2 Univariate and Multivariate Logistic Regression

**FIGURE 2:**
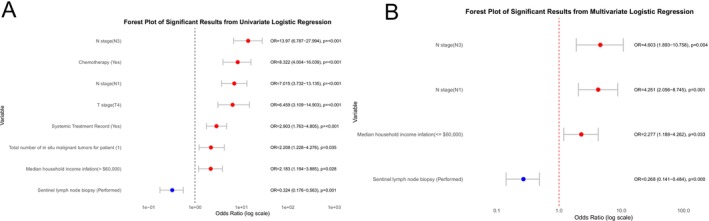
Forest plot of statistically significant variables identified in the univariate (A) and multivariate (B) logistic regression analyses for distant metastasis in patients with acral melanoma. Only variables with statistical significance in each model are shown.

In the univariate LR analysis, undergoing sentinel lymph node biopsy was identified as a protective factor against distant metastasis in AM patients (odds ratio [OR] < 1, 95% confidence interval [CI] < 1, *p* < 0.05). On the contrary, chemotherapy, systemic therapy, higher T stage, higher N stage, greater total number of in situ malignant tumors, and lower median household income were determined to be risk factors for distant metastasis (OR and 95% CI > 1, *p* < 0.05). In the multivariate LR analysis, sentinel lymph node biopsy remained an independent protective factor against distant metastasis (OR and 95% CI < 1, *p* < 0.05). Additionally, the multivariate analysis demonstrated that having a higher N stage and having a lower median household income were independent risk factors for distant metastasis in AM patients (OR and 95% CI > 1, *p* < 0.05) (Table 3). Figure 2A,B presents the forest plot corresponding to the statistically significant results from the univariate and multivariate LR analyses.

**TABLE 3:** Univariate and multivariate logistic regression.

Variable	Factor	Univariate analysis		Multivariate analysis	
		OR (95% CI)	*p*	OR (95% CI)	*p*
Age	> 60	Ref	Ref	Ref	Ref
	≤ 60	1.476 (0.897–2.449)	0.2	\	\
Sex	Female	Ref	Ref	Ref	Ref
	Male	1.499 (0.910–2.500)	0.185	\	\
Race	Black	Ref	Ref	Ref	Ref
	Others (American Indian/Alaska Native, Asian or Pacific Islander)	1.352 (0.466–4.233)	0.646	\	\
	White	0.806 (0.363–2.181)	0.686	\	\
Total number of in situ malignant tumors	> 1	Ref	Ref	Ref	Ref
	1	2.208 (1.228–4.276)	0.035	1.612 (0.855–3.239)	0.235
Rural–urban continuum code	Metropolitan	Ref	Ref	Ref	Ref
	Nonmetropolitan	1.588 (0.787–2.938)	0.244	\	\
Median household income inflation adj to 2022	$60 001–$89 999 (reference)	Ref	Ref	Ref	Ref
	≤ $60 000	2.183 (1.194–3.885)	0.028	2.277 (1.188–4.262)	0.033
	≥ $90 000	0.950 (0.489–1.757)	0.895	0.875 (0.429–1.701)	0.749
Time from diagnosis to treatment	≤ 7 days	Ref	Ref	Ref	Ref
	31–90 days	0.649 (0.304–1.259)	0.312	\	\
	8–30 days	0.395 (0.145–0.883)	0.084	\	\
	Over 91 days	1.609 (0.364–4.703)	0.526	\	\
	Unable to calculate	0.790 (0.290–1.777)	0.663	\	\
Primary site	Skin of upper limb and shoulder	Ref	Ref	Ref	Ref
	Skin of lower limb and hip	1.488 (0.758–3.314)	0.369	\	\
Sentinel lymph node biopsy record	Not performed	Ref	Ref	Ref	Ref
	Performed sentinel biopsy	0.324 (0.176–0.563)	0.001	0.268 (0.141–0.484)	< 0.001
Chemotherapy recode	No	Ref	Ref	Ref	Ref
	Yes	8.322 (4.004–16.039)	< 0.001	1.985 (0.827–4.503)	0.18
Systemic therapy recode	No	Ref	Ref	Ref	Ref
	Yes	2.903 (1.763–4.805)	< 0.001	1.646 (0.903–2.967)	0.166
Surgery record	No	Ref	Ref	Ref	Ref
	Yes	0.347 (0.140–1.123)	0.087	\	\
T stage	T1	Ref	Ref	Ref	Ref
	T2	2.584 (1.092–6.434)	0.074	2.394 (0.964–6.2)	0.118
	T3	2.668 (1.153–6.553)	0.059	1.844 (0.725–4.886)	0.286
	T4	6.459 (3.109–14.903)	< 0.001	2.68 (1.138–6.781)	0.067
N stage	N0	Ref	Ref	Ref	Ref
	N1	7.015 (3.732–13.135)	< 0.001	4.251 (2.056–8.745)	< 0.001
	N2	3.842 (1.621–8.330)	0.006	2.464 (0.985–5.722)	0.088
	N3	13.970 (6.787–27.994)	< 0.001	4.603 (1.893–10.758)	0.004

### 3.3 Model Performance

**FIGURE 3:**
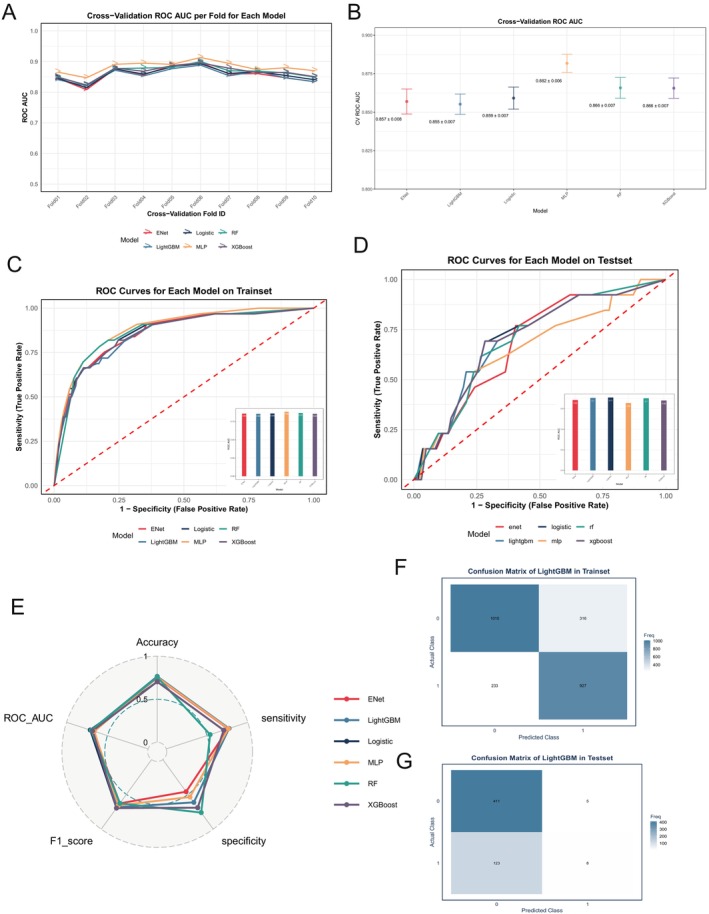
Performance of the six machine learning models for predicting distant metastasis in acral melanoma. (A) ROC AUC values from 10‐fold cross‐validation for each model, showing relatively stable performance across folds. (B) Mean ROC AUC values with standard deviations obtained from 10‐fold cross‐validation for each model. (C) ROC curves of the six models in the training set; the inset bar plot shows the corresponding AUC values. (D) ROC curves of the six models in the internal test set; the inset bar plot shows the corresponding AUC values. (E) Radar plot comparing model performance across multiple metrics, including accuracy, sensitivity, specificity, F1 score, and ROC AUC. (F) Confusion matrix of the LightGBM model in the training set. (G) Confusion matrix of the LightGBM model in the internal test set.

Based on the results of the multivariable logistic regression analysis after excluding treatment‐related variables from the model, three variables were identified with statistically significant associations (*p* < 0.05): sentinel lymph node biopsy, N stage, and median household income. These variables were subsequently incorporated as features in the machine learning model. We developed predictive models for AM metastasis via six machine learning algorithms: logistic regression, ENet, XGBoost, MLP, RF, and LightGBM. The discriminatory ability of the models was evaluated using the area under the receiver operating characteristic (ROC) curve (AUC). During 10‐fold cross‐validation, the AUC values of the six models remained relatively stable across different folds, with no substantial fluctuations observed, suggesting relatively consistent performance across folds during internal validation (Figure 3A). The aggregated cross‐validation results showed that all six models achieved relatively high mean AUC values, suggesting that each model possessed moderate discriminative ability in distinguishing patients with and without distant metastasis. Among them, ENet and LightGBM exhibited comparatively higher AUC values, whereas the overall performance of the MLP model was relatively lower (Figure 3B).

ROC curves were further generated for the training and test sets to assess model generalizability. As shown in Figure 3C,D, all six models demonstrated reasonable discriminative performance in both datasets, with ROC curves clearly exceeding the reference line of random classification. All six models showed measurable predictive performance. The radar chart in Figure 3E provides a comprehensive comparison of model performance across multiple metrics, including accuracy, F1 score, sensitivity, specificity, and AUC. The detailed evaluation metrics of the six machine learning algorithms in the internal test set are summarized in Table 4. The calibration curves corresponding to the six models are shown in the Figure S1. The results indicated that LightGBM exhibited a relatively balanced performance across all metrics, with a larger overall coverage area, suggesting a more balanced overall performance. In contrast, although other models showed advantages in specific individual metrics, their overall performance was less balanced. Collectively, these findings suggested that LightGBM demonstrated a more robust overall performance in multi‐metric evaluation. Given the high degree of class imbalance, multiple complementary performance metrics were jointly used to provide a comprehensive evaluation and to reduce potential bias associated with reliance on a single metric. Figure 3F,G presents the confusion matrices of the LightGBM model for the training and test sets, respectively.

**TABLE 4:** Comparison of evaluation metrics for various machine learning algorithms in the internal test set.

Model	Accuracy	Specificity	Sensitivity	F1_score	ROC_AUC
Logistic	0.704	0.692	0.704	0.697	0.71
ENet	0.751	0.462	0.758	0.629	0.68
XGBoost	0.704	0.692	0.704	0.697	0.68
MLP	0.757	0.538	0.762	0.659	0.66
LightGBM	0.766	0.615	0.77	0.686	0.7
RF	0.757	0.762	0.538	0.629	0.7

### 3.4 Feature Importance and Visualization of the LightGBM Model

**FIGURE 4:**
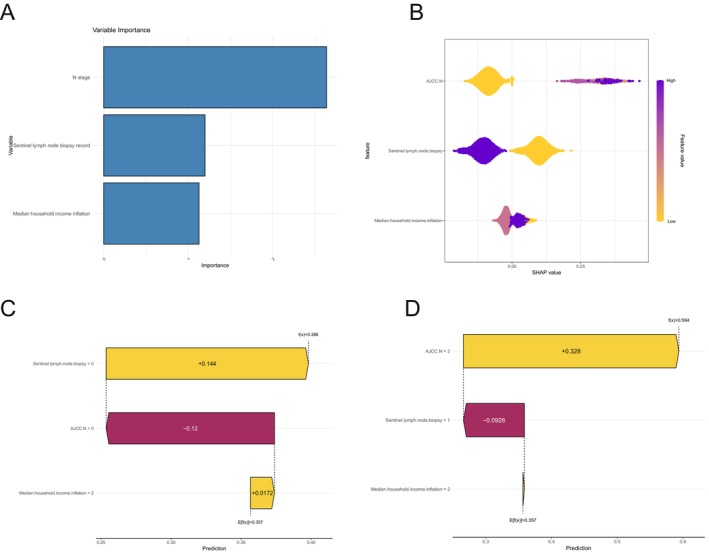
SHAP model explanation for feature variables with LightGBM machine learning model. (A and B) Features of distant metastasis of patients with acral melanoma based on the importance ranking of feature variables in the LightGBM machine learning model. (C and D) Distribution of SHAP values for two patients. Positive SHAP values (indicated in yellow) represent risk factors for distant metastasis in AM patients, whereas negative SHAP values (indicated in purple) represent protective factors. Median household income inflation (“≤ $60 000” = 0, “$60 000–$89 999” = 1, and “> $90 000” = 2); N stage (0 = N0, 1 = N1, 2 = N2, and 3 = N3); and Sentinel lymph node biopsy (“Yes” = 1 and “No” = 0).

Given the superior performance of the LightGBM model, we generated a feature importance ranking with the help of SHAP values. The top‐ranked features contributing to the likelihood of distant metastasis in AM patients were N stage, sentinel lymph node biopsy record, and median household income inflation (Figure 4A,B). Two waterfall plots were subsequently generated to show the feature interpretation of the predictions for individual samples (Figure 4C,D). In the figure, yellow indicates risk factors for distant metastasis in patients with AM, whereas purple indicates protective factors.

### 3.5 Online Web Calculator

**FIGURE 5:**
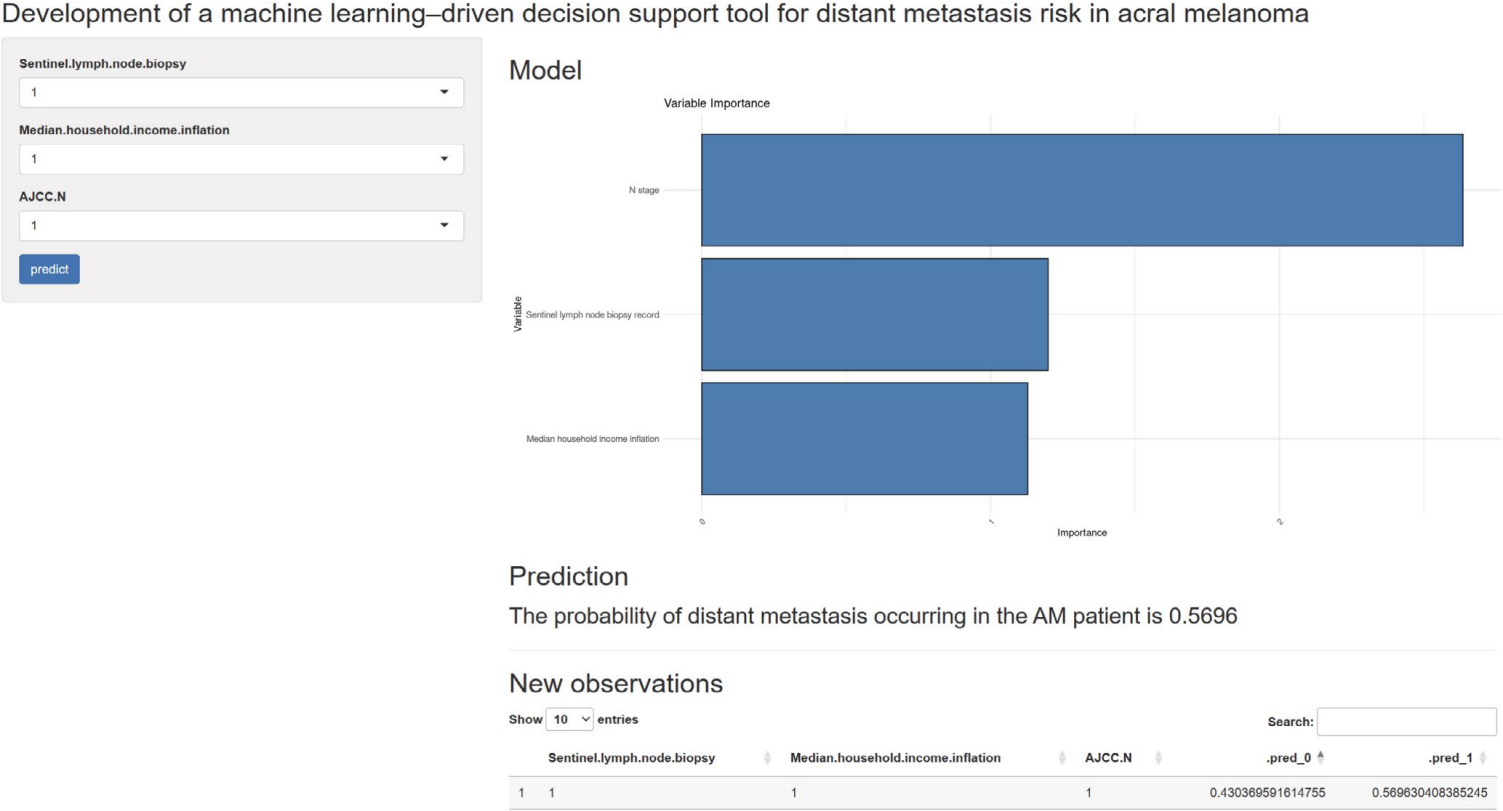
Web‐based calculator developed from the LightGBM model for research‐oriented visualization of individualized distant metastasis risk in acral melanoma. Median household income inflation (“≤ $60 000” = 0, “$60 000–$89 999” = 1, and “> $90 000” = 2); N stage (0 = N0, 1 = N1, 2 = N2, and 3 = N3); and Sentinel lymph node biopsy (“Yes” = 1 and “No” = 0).

Using the optimal LightGBM model, we developed a web‐based calculator for individualized risk assessment. Users can enter the values of the predictor variables on the left side of the webpage, and the calculator provides the estimated probability of distant metastasis in real time (https://yeshanyuan.shinyapps.io/yeshanyuan/; Figure 5).

## 4 Discussion

AM is associated with high mortality and poor survival rates [21] and has a worse prognosis than other melanoma subtypes [22, 23]. Distant metastasis, which commonly affects organs such as the lungs, liver, and brain, is a major contributor to the poor prognosis of patients with AM. The median survival time after distant metastasis is generally short, approximately 11–12 months [24]. Notably, metastatic lesions can develop directly from the primary tumor without necessarily involving local lymph node spread [25]. At the same time, early micro metastases, which are clinically undetectable, significantly increase the likelihood of distant metastasis in AM patients, reducing their OS time [6]. This underscores the critical importance of predicting the risk of distant metastasis at an early stage. However, relevant research is still scarce and faces several challenges. For example, research on metastatic AM has relied predominantly on retrospective cohort studies, which often lack detailed patient‐specific prognostic factors and comprehensive treatment plans. To overcome this limitation, we utilized the comprehensive patient data in the SEER database to retrospectively collect clinical, baseline, and therapy information.

In the past, scholars analyzed three gene expression datasets and developed a support vector machine (SVM) classifier to predict cutaneous melanoma metastasis. The model identified 110 key genes and achieved prediction accuracies of 96.8%, 100%, and 94.4% across the datasets [26]. This study, however, has notable weaknesses. First, although assessing clinical data of AM patients is necessary for their prognosis, there is no comparison of baseline data or evaluation of the effect of each treatment modality on developing distant metastasis. Additionally, the limited accessibility of gene expression data in clinical settings constrains the model's practical application. Nevertheless, its clinical utility could be further enhanced by integrating patients' baseline clinical information and historical treatment data. In this study, we used the SEER database to collect clinical data on AM patients, including primary tumor location, stage, treatment methods, and baseline characteristics. The study's large sample size and long‐term follow‐up data support data‐intensive approaches like machine learning. Although we cannot directly determine whether AM patients have micrometastases or undetectable distant metastases through routine clinical examinations, predictive models can estimate the probability of distant metastasis based on the patient's baseline data.

Given the pronounced class imbalance inherent in distant metastasis outcomes, an oversampling strategy was applied during model training to mitigate bias introduced by skewed class distributions, while evaluation relied on multiple complementary performance metrics to avoid overinterpretation of any single indicator. The current findings should be interpreted as proof of concept, and external validation using independent real‐world cohorts will be essential before routine clinical implementation.

Our research also suggested that sentinel lymph node biopsy was a protective factor against distant metastasis. Numerous studies have shown that sentinel lymph node biopsy is a strong predictor of disease‐free survival, disease‐specific survival, and OS [27, 28, 29, 30]. In clinical practice, our findings further highlighted the potential relevance of sentinel lymph node biopsy in risk stratification for AM patients. Therefore, accurately assessing the impact of these factors on the occurrence of distant metastasis in AM patients and promptly implementing warning systems and relevant interventions is crucial for improving the long‐term survival prognosis of AM patients. Regarding the impact of income on metastasis and prognosis in melanoma patients, Hernandez et al. reported that patients in rural, low‐income areas have lower surgery rates and poorer melanoma‐specific survival due to limited access to medical resources [31]. Similarly, Joshi et al. found that patients from low‐income households are more likely to be diagnosed at a later stage and experience significantly lower OS compared to those from higher‐income households [32]. These findings were consistent with our model, where “median household income ≤ $60 000” was identified as a risk factor for distant metastasis in patients. Therefore, bridging the income gap and ensuring greater access to quality healthcare in relatively impoverished areas remain urgent issues that must be addressed.

In our current research, we developed six machine learning models using the feature variables mentioned above and identified LightGBM as the most effective. LightGBM is an efficient gradient boosting decision tree with key advantages such as fast training speed and excellent scalability. It can handle large‐scale data and high‐dimensional features while maintaining high accuracy. By incorporating techniques like gradient‐based one‐sided sampling (GOSS) and exclusive feature bundling (EFB), LightGBM significantly reduces computational complexity, lowers memory usage, and natively handles categorical features, making it well‐suited for complex, large datasets [33]. Among the evaluated algorithms, LightGBM was ultimately selected as the primary modeling approach based on its overall robustness and suitability for the characteristics of the present dataset. First, LightGBM demonstrated more balanced and stable performance across multiple evaluation metrics, including AUC, precision, and F1 score [34]. This consistency is particularly important in the context of distant metastasis, which represents a low‐incidence clinical outcome in AM, where reliance on a single metric may lead to misleading conclusions. Second, as a gradient boosting decision tree–based method, LightGBM is well‐suited for capturing complex nonlinear relationships and feature interactions among multidimensional clinical variables. Such modeling flexibility is essential for oncological risk prediction, where disease progression is rarely governed by linear effects alone [13]. Third, when combined with synthetic minority over‐sampling techniques (SMOTE), LightGBM maintained strong discriminative performance under conditions of severe class imbalance. This indicates its adaptability to imbalanced clinical datasets and supports its application in rare‐event prediction scenarios. Finally, LightGBM offers a distinct advantage in terms of interpretability when integrated with SHAP‐based explanation frameworks [35]. This compatibility enables both global feature importance analysis and individualized prediction interpretation, thereby enhancing the transparency and clinical acceptability of the model. Collectively, these strengths justify the selection of LightGBM as a suitable and reliable approach for distant metastasis risk prediction in AM. Given the pronounced class imbalance in distant metastasis, an oversampling strategy was applied during model training to mitigate class distribution bias, while multiple complementary performance metrics were jointly used during evaluation to avoid overreliance on any single metric. We subsequently developed an online web calculator based on the LightGBM machine learning model. The web‐based calculator developed in this study is primarily intended for research‐oriented risk stratification and methodological demonstration, aiming to intuitively illustrate the potential application of the machine learning model in individualized risk assessment, rather than serving as a clinical decision‐making tool. To highlight the importance of the variables, we applied the SHAP method for a global explanation of the model features.

The present study has several methodological strengths. First, a large‐scale real‐world cohort with long‐term follow‐up was constructed from the SEER database, enabling modeling of distant metastasis, a low‐incidence outcome in AM. Second, multiple machine learning algorithms were systematically compared, and LightGBM was selected for its balanced performance and robustness under class imbalance. Finally, SHAP‐based interpretability and a web‐based calculator were incorporated to enhance model transparency and research‐oriented risk stratification.

Despite the encouraging results, several limitations should be acknowledged. First, this study was based on a retrospective registry‐based cohort, and the number of distant metastasis events was very small (45 out of 1822 patients, approximately 2.5%), which may have affected model stability and the reliability of performance estimates. Although oversampling, cross‐validation, and independent test set evaluation were applied to reduce the impact of class imbalance, these approaches can only partially mitigate the statistical instability and potential risk of overfitting associated with low‐event settings. In addition, this study was conducted using a retrospective registry‐based cohort, and the model has not yet been externally validated using independent real‐world datasets. Therefore, the current findings should be interpreted as proof of concept, and external validation is required before routine clinical implementation. Second, the proposed model was developed exclusively using structured clinical and pathological variables from the SEER database and did not incorporate dermoscopic or histopathological imaging data [36, 37]. The integration of imaging or multimodal data may further improve risk stratification and predictive performance and should be explored in future studies.

## 5 Conclusion

Overall, the LightGBM model demonstrated moderate predictive performance for distant metastasis risk stratification in patients with AM. It may serve as a research‐oriented tool for individualized risk assessment, pending further external validation before clinical application.

## Author Contributions

**Lv Qun:** methodology, validation. **Zhang Qian:** methodology, validation. **Ye Shanyuan:** conceptualization, investigation, methodology, software, validation. **Zhang Rundong:** conceptualization, investigation, validation, methodology. **Cao Meng:** conceptualization, investigation. **Qiu Zequn:** methodology, validation. **Wang Yan:** methodology, validation.

## Funding

This work was supported by the National Key Research and Development Program of China (2022YFC2504700, 2022YFC2504701, 2022YFC2504705), the Medical Scientific Research Project of Jiangsu Provincial Health Commission (Grant No. M2024012), the National Natural Science Foundation of China (NSFC; 81872216), and the CAMS Innovation Fund for Medical Sciences (2024‐I2M‐C&T‐B‐089).

## Ethics Statement

The National Cancer Institute deemed this cohort study to be exempt from institutional review board approval because publicly available de‐identified data were used. The study followed the Strengthening the Reporting of Observational Studies in Epidemiology (STROBE) reporting guideline.

## Consent

The authors have nothing to report.

## Conflicts of Interest

The authors declare no conflicts of interest.

## Supporting information


**Figure S1:** Calibration curves of the six machine learning models for predicting distant metastasis in acral melanoma.

## Data Availability

The datasets analyzed for this study can be found in the SEER database. Please see https://seer.cancer.gov/ for more details.
