# Supplementary figures and images for: Development of a Machine Learning Model for Distant Metastasis Risk Stratification in Acral Melanoma
Source: Cancer Rep (Hoboken). 2026 May 24;9(5):e70569. doi: 10.1002/cnr2.70569 (PMC13240029; doi:10.1002/cnr2.70569)

# Calibration Curves for Each Model

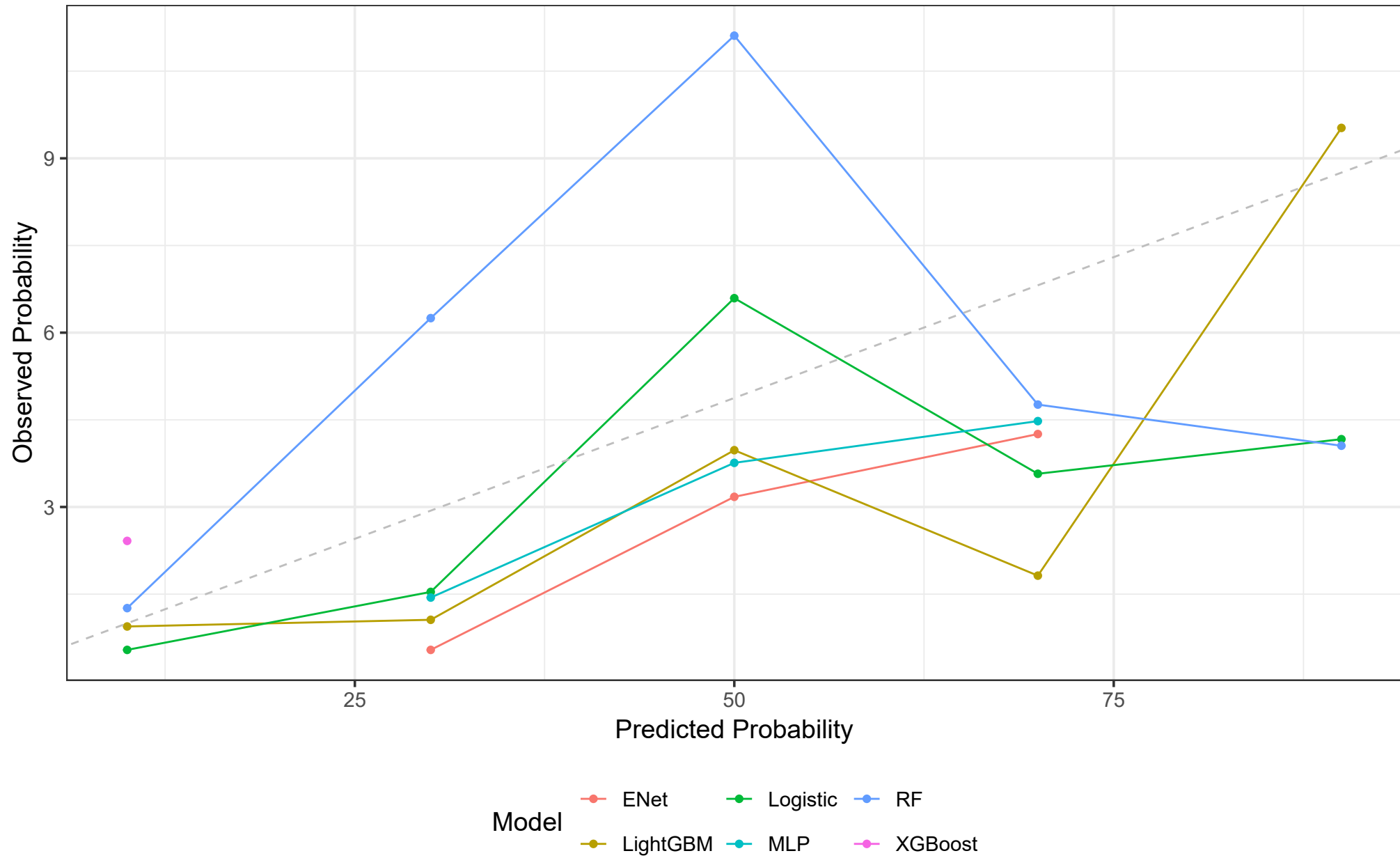

Supplement: Supplementary file 1 — Figure S1: Calibration curves of the six machine learning models for predicting distant metastasis in acral melanoma. [file CNR2-9-e70569-s001.pdf]
